# Effect of dipterinyl calcium pentahydrate on hepatitis B virus replication in transgenic mice

**DOI:** 10.1186/1479-5876-8-32

**Published:** 2010-03-31

**Authors:** Phillip Moheno, John Morrey, Dietmar Fuchs

**Affiliations:** 1 SanRx Pharmaceuticals, Inc., 603 Colima Street, La Jolla, California 92037-8032, USA; 2 Institute for Antiviral Research, 4700 Old Main Hill, Biotechnology Center, Utah State University, Logan, Utah 84322-4700, USA; 3 Division of Biological Chemistry, Biocentre, Innsbruck Medical University, Fritz Pregl Strasse 3, A-6020 Innsbruck, Austria

## Abstract

**Background:**

Dipterinyl calcium pentahydrate (DCP) has previously been shown to inhibit MDA-MB-231 human breast cancer xenographs in nude mice in a manner correlated with increases in plasma IL-12 and IL-4 concentrations, and decreases in plasma IL-6 levels. DCP also inhibits indoleamine 2,3-dioxygenase (IDO), an immuno-inhibitory enzyme, in human PBMCs (Peripheral Blood Mononuclear Cells).

**Methods:**

In the present study, DCP was administered per os, once daily for 14 days to hepatitis B virus (HBV) transgenic mice at 23, 7.3, and 2.3 mg/(kg d). Multivariate stepwise regression and MANOVA analyses, by gender and treatment, of liver HBV DNA and RNA measures, liver core and serum HBe antigen assays, serum cytokine/chemokine profiles, and IDO metabolite measurements were performed.

**Results:**

DCP caused a significant dose-response reduction of log liver HBV DNA as measured by PCR in the female HBV mice. The gender dependence of the anti-HBV DNA activity was explained by the DCP Effects Model (DCP-EM) (*p *= .001) which includes three serum biomarker changes caused by DCP: 1) decreased MCP-1; 2) decreased Kyn/Trp (an estimation of IDO activity); and 3) increased GM-CSF.

**Conclusions:**

Immunomodulation via IDO or TDO (tryptophan 2,3-dioxygenase) pathways, along with serum MCP-1 and GM-CSF are proposed to play roles in the anti-HBV mechanism of DCP based upon their coordinated modulation in the reduction of viral DNA replication in HBV mice.

## Background

**Figure 1:**
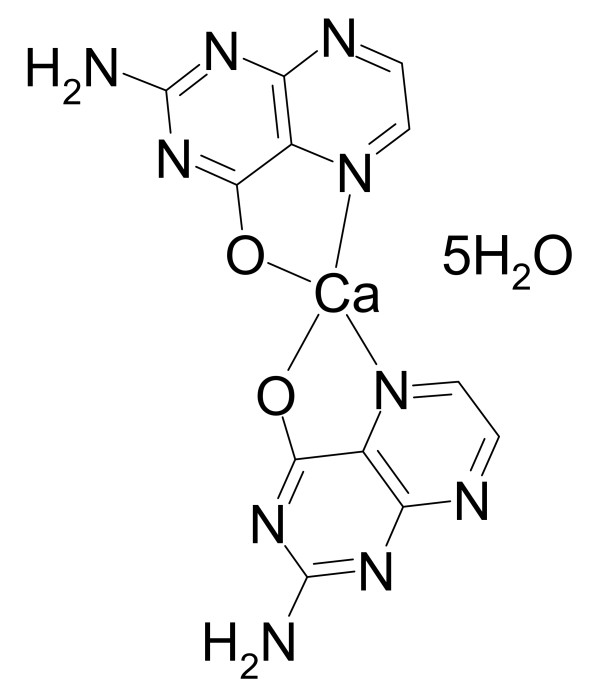
**The structure of dipterinyl calcium pentahydrate, (C_6_H_4_N_5_O)_2_Ca·5H_2_O (MW 454.4)**. The X-ray crystallographic structure was given previously [3].

Hepatitis B virus (HBV) causes both transient and persistent infections of the liver in humans. The number of chronic HBV carriers is estimated to be 400 million worldwide; nearly 25% of which are projected to succumb to liver failure or liver cancer [1]. Additionally, HBV infection remains an important cause of acute and chronic liver disease in the United States [2]. Dipterinyl calcium pentahydrate (DCP), shown in Figure 1, has demonstrated significant antitumor activity associated with plasma IL-12 concentration increases in MDA-MB-231 (human breast cancer) xenographs in nude mice [3,4]. This finding, along with previous work demonstrating IL-12 suppression of HBV replication in transgenic mice [5], prompted us to investigate the activities of DCP in the HBV transgenic mouse model.

DCP is a stable, sparingly soluble compound that can be solubilized in aqueous solutions to 440 μM with sonication. For this study, an orally administered suspension was used. It was hypothesized that because of the antitumor changes elicited by DCP, as well as the anti- and pro-inflammatory plasma cytokine/chemokine concentration changes reported previously, DCP would reduce liver HBV DNA levels and possibly other HBV parameters in transgenic mice carrying an infectious clone of HBV. The investigators anticipated that DCP might work via cytokine/chemokine modulatory mechanisms similar to those described by others [5-9].

Evidence for immunomodulation by DCP was further investigated by measurement and analysis of the enzyme indoleamine 2,3-dioxygenase (IDO) serum metabolites, tryptophan (Trp) and kynurenine (Kyn). Tryptophan (Trp) is the substrate for IDO, a key immunological inhibitor of T cells, and an identified tumor escape mechanism [10]. Recent studies have demonstrated that IDO-mediated immune suppression is prevalent in hepatitis B infection [11]. The IDO enzymatic product, kynurenine (Kyn), and its downstream metabolites, kynurenine, 3-hydroxykynurenine, and 3-hydroxyanthranilic acid, are directly involved in the regulation of T cells and other lymphocytic cell types, *i.e*., NK cells and B cells [12]. We have shown previously that DCP inhibits IDO activity in human PBMCs (Peripheral Blood Mononuclear Cells) [3]. Certain neurotoxic end-products of the tryptophan-kynurenine pathway, such as quinolinic acid, produced under chronic inflammatory conditions (e.g., cardiovascular disease, multiple sclerosis, diabetes, cancer, and major depression) may contribute to the brain damage seen in depression and dementia [13]. For the study described here, the calculation of the serum Kyn-to-Trp ratio (Kyn/Trp) allowed us to estimate the extent of IDO activity in the serum of the HBV mice [14].

## Materials and methods

### Materials

#### Compounds

DCP was suspended in 0.4% carboxymethylcellulose (CMC) at concentrations sufficient to deliver the desired dose by oral gavage in a volume of 0.1 mL per 20 g mouse. The solution was stored at 4°C during the course of the experiment. The volume was adjusted for the weight of each mouse. The structure of DCP [3] is given in Figure 1. The positive control, adefovir dipivoxil (ADV) (Gilead Pharmaceuticals), was prepared in the same manner as the DCP for the appropriate dosages.

### Methods

#### In vivo testing

##### Animals

Homozygous adult female and male transgenic HBV mice were used (20.6 ± 2.8 g). The mice were originally obtained from Dr. Frank Chisari (Scripps Research Institute, La Jolla, CA) [15] and were subsequently raised in the Biosafety Level 3 (BL-3) area of the AAALAC-accredited USU Laboratory Animal Research Center (LARC). The animals were derived from founder 1.3.32 [15]. This study was conducted in accordance with the approval of the Institutional Animal Care and Use Committee of Utah State University.

##### Experimental design

DCP was administered per os, once daily for 14 days to 94 randomly assigned HBV transgenic mice at 23 mg/(kg d) (10 females/10 males), 7.3 mg/(kg d) (10 females/10 males), and 2.3 mg/(kg d) (10 females/10 males). DCP dosages were based upon previously reported efficacious levels for antitumor activity [3]. ADV was used as a positive control at 10 mg/(kg d) (10 females/5 males) using the same treatment schedule and vehicle (0.4% CMC). 10 female/9 male control vehicle mice were used. This efficacious ADV dosage used was previously demonstrated in HBV mice [9]. On day 14, mice were euthanized to collect serum and liver samples to perform liver HBV DNA, liver HBV RNA, liver core and serum HBe antigen assays, serum cytokine/chemokine profiles, and IDO metabolite measurements. No plasma level measurements of DCP were performed.

#### Virology

##### Liver HBV DNA assays

Southern blot hybridization and quantitative PCR (qPCR) were performed on liver tissues [9]. For Southern blot hybridization, the ratio of the viral DNA bands to the transgene band was used to determine the concentration of viral DNA per host DNA. This calculation was based upon the knowledge that there were 1.3 copies of the transgene present per host cell with this line of transgenic mice. The transgene was used as an internal indicator to calculate the pg of HBV DNA per μg of homozygous cellular host DNA. For qPCR, the assay was run with a series of 10-fold dilutions of pooled liver DNA from HBV transgenic mice to obtain a standard curve. Mean C(t) values were obtained for duplicates of each sample. The mean C(t) values of each sample were used to obtain the log relative DNA values using a formula of the fit line of the standard curve.

##### Liver HBV RNA

Liver samples were assayed for HBV RNA as described previously [9]. The RT-PCR technique used for determining liver HBV DNA (PCR) has been reported to be more rapid and sensitive, and can be more specific that northern blot analysis [16].

##### Liver or serum cytokine/chemokine array

Liver samples were prepared for the Q-Plex™ mouse cytokine/chemokine array (Quansys Biosciences, Logan, UT) as described previously [9].

##### Sera HBeAg

Whole blood samples were obtained during necropsy by cardiac puncture, and processed for HBeAg-specific ELISA (International Immuno Diagnostics, Foster City CA) [9]. Using the known PEI (Paul Ehrlich Institute) units for the calibrator, PEI units were formulated for the serial dilutions of the positive serum. A graph was generated, and extrapolation was used to assign a PEI unit value for each sample.

##### Liver HBcAg assay

Liver biopsies were processed for detection of hepatitis B core antigen (HBcAg) [9]. Three different parameters were obtained from each tissue section. The first two measurements were based on the observation that cells surrounding the central veins of the liver are more strongly stained than are other areas of the liver (personal observation). The first two parameters were obtained by counting cells surrounding central veins as follows: the total number of cells, the number of cells with stained nuclei, and the number of cells with stained cytoplasms. The identities of the samples were blinded to the person counting. The stained nuclei counts or the stained cytoplasm counts were divided by the total number of cells. Three central vein areas were counted for each slide sample. For the third parameter, a field not in a central vein area was counted for the total number of stained nuclei. One-quarter of the field was counted. Three such fields were counted per liver section. The identities of the samples were blind to the person reading the slides.

##### Tryptophan and kynurenine measurements

Serum tryptophan (Trp) and kynurenine (Kyn) measurements were carried out as previously described [17]. Kyn/Trp ratios were calculated for each mouse as an estimate of IDO activity.

### Statistical analysis

Those measures found to be significant by the Kruskal-Wallis nonparametric test for treatment group effects were then tested by one-way ANOVA, followed by post-hoc 2-sided Dunnett tests (for equal variances) or Dunnett's T3 tests (for unequal variances) versus controls. The Mann-Whitney U nonparametric test was used to test gender effects, followed by one-way ANOVA. A 2-way MANOVA was carried out to test gender-treatment interactions. Multivariate stepwise regression analysis was performed on the full data set, along with six log transformed DNA and RNA variables, to identify a significant, small-number cluster of variables modeling DCP dosage from the larger number of variables measured. SPSS Graduate Pack 15.0 for Windows (2006) was utilized with *p *< .05 used to determine significance.

## Results

The following viral, IDO, and cytokine/chemokine measures were collected in this study: liver HBV DNA (Southern), liver HBV DNA (PCR), liver HBV RNA (PCR), HBe antigen (ELISA); Average # HBcAg Nuclei, Average # HBcAg Cytoplasms, # HBcAg Nuclei per Quarter Field; serum Tryptophan, Kynurenine, Kyn/Trp, IL-1a, IL-1b, IL-2, IL-3, IL-4, IL-6, IL-9, IL-10, IL-12, MCP-1, TNF-α, MIP-1, GM-CSF, RANTES; and liver IL-6. See additional file 1: "Summary Statistics" for the data summary and statistical results from this study.

Table 1, Table 2 gives the derivation of the significant (*p *= .001) DCP Effects Model (DCP-EM) variable cluster from the linear multivariate stepwise regression of DCP dosage versus all the viral, IDO, and cytokine/chemokine measures. The logarithmically transformed liver HBV DNA (Southern), liver HBV DNA (PCR), liver HBV RNA (PCR), and HBe antigen (ELISA) measures were included in this analysis as in previous studies [9,18,19]. The DCP Effects Model linear stepwise regression analysis identified a significant (p = .001) cluster of variables responding to the DCP treatment. Stepwise regression analyses, in general, derive a small cluster of variables from a much larger set of variables in order to model a particular variable of interest, in the present study, the DCP dose. Taken individually, the component variables of a derived cluster do not achieve the same level of significance as the cluster taken as a whole. In the present study, Log liver HBV DNA (PCR) emerged as the single strongest viral variable which significantly and inversely modeled DCP dosage. None of the other viral measures significantly further explained the DCP dosage variance. These other viral measures included: 1) liver HBV DNA (Southern), 2) liver HBV DNA (PCR), 3) liver HBV RNA (PCR), 4) the log liver HBV DNA (Southern), and 5) log liver HBV RNA (PCR). The DCP Effects Model regression, however, identified the serum measures Kyn/Trp, MCP-1, and GM-CSF as significant additional variables which further accounted for DCP dosage variance (Table 1, Table 2).

**Table 1:** The DCP Effects Model (DCP-EM) was derived from the stepwise regression of the DCP dose versus: serum Tryptophan, Kynurenine, Kyn/Trp; HBV DNA [Southern], HBV DNA [PCR], HBV RNA [PCR], HBe antigen [ELISA]; Average # Liver HBcAg Nuclei per Total, Average # Liver HBcAg Cytoplasms per Total, Average # Liver HBcAg Nuclei per Quarter Field; IL-1a, IL-1b, IL-2, IL-3, IL-4, IL-6, IL-9, IL-10, IL-12, MCP-1, TNF-α, MIP-1, GM-CSF, RANTES, and liver IL-6; log HBV DNA [Southern], log rel. HBV DNA [PCR], log HBV RNA [PCR], and log HBe antigen [ELISA].

Coefficients of variables in cluster modelling DCP dosing effects				
	**Unstandardized Coefficients**		**Standardized Coefficients**	
	
DCP-EM Model* (variable cluster)	**B**	**Std. Error**	**Beta**	**Sig**.

(Constant)	26.309	4.976		.000
MCP-1	-.022	.007	-.371	.003
Log HBV DNA (PCR)	-4.065	1.376	-.359	.005
Kyn/Trp	-.560	.196	-.349	.006
GM-CSF	.070	.030	.283	.023

Probability-to-Enter = .10 and Probability-to-Remove = .15. The model is significant to *p = .001.

Derived DCP-EM Regression Model*:

DCP-EM score = 26.309 - 0.22 [MCP-1 rel. pg/ml] - 4.065 [Log rel. HBV DNA (PCR)]

-.560 [Kyn/Trp uM/mM] + 0.070 [GM-CSF rel. pg/ml]

**Table 2:** Excluded variables not included in DCP-EM model

Model	Beta In	**Sig**.	Partial Correlation
Trp	.097	.453	.100
Kyn	-.008	.977	-.004
HBV DNA (Southern)	-.013	.932	-.011
HBV DNA (PCR)	-.130	.491	-.091
HBV RNA (PCR)	-.008	.947	-.009
HBe (ELISA)	.187	.184	.175
Ave Liver HBcAg Nuclei per Total	.040	.739	.044
Ave Liver HBcAg Cytoplasm per Total	.100	.399	.112
Ave Liver HBcAg Nuclei per Quarter Field	-.051	.698	-.052
IL-1a	-.073	.531	-.083
IL-1b	-.058	.639	-.062
IL-2	-.112	.534	-.083
IL-3	-.112	.336	-.128
IL-4	.157	.175	.179
IL-6	-.128	.282	-.142
IL-9	.043	.718	.048
IL-10	-.089	.447	-.101
IL-12	-.023	.850	-.025
TNF-α	-.078	.532	-.083
MIP-1	.047	.690	.053
RANTES	.166	.161	.185
Liver IL-6	.057	.626	.065
Log HBV DNA (Southern)	.044	.804	.033
Log HBe (ELISA)	.178	.176	.178
Log HBV RNA (PCR)	-.011	.925	-.012

The molecular biology of HBV RNA synthesis, which was not affected by any of the treatments in the HBV transgenic mice, is significantly different from that of the natural HBV infection.

The HBV RNA in the transgenic mice is produced primarily from an HBV transgene, unlike a natural infection wherein it is expressed from HBV covalently closed circular DNA (cccDNA) (Raney et al., 2001) [20]. Unlike the natural infection, HBV produced from the transgene in mice cannot infect mouse cells for successive rounds of viral replication. In the natural infection, HBV RNA and HBe and HBs proteins are derived from successive rounds of replication to produce increasing levels of cccDNA, and consequently, increasing levels of HBV RNA and proteins. In this regard the use of the HBV transgenic mice is analogous to HBV stably transfected, cells such as Hep G 2.2.15 cells (Iyer et al., 2004a) [21]. It was not unexpected, therefore, that a selective reduction of HBV DNA would not necessarily affect HBV RNA or protein levels, since these levels can be independently derived from the HBV RNA transcribed from the transgene. [9]

**Figure 2:**
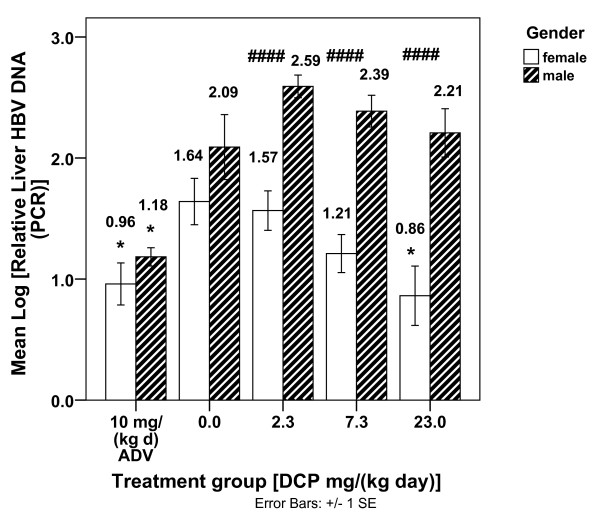
**The Mean Log [relative Liver HBV DNA (PCR)] by treatment group and gender for HBV mice**. DCP was administered per os, once daily for 14 days to 94 randomly assigned HBV transgenic mice at 23 mg/(kg d) (10 females/10 males), 7.3 mg/(kg d) (10 females/10 males), and 2.3 mg/(kg d) (10 females/10 males). ADV was used as a positive control at 10 mg/(kg d) (10 females/5 males) using the same treatment schedule and vehicle (0.4% CMC). 10 female/9 male control vehicle mice were used. Mean Log [relative Liver HBV DNA (PCR)] values, as measured by quantitative PCR are graphed by treatment group and gender for HBV mice (ADV = Adefovir dipivoxil). * (*p *< .05) - Mean Log [relative liver HBV DNA (PCR)] is significantly different than controls [DCP = 0.0 mg/(kg d) of the same gender] for females at 23.0 mg/(kg d) DCP and for ADV in both females and males. ^#### ^(*p *≤ .001) - Mean Log [relative liver HBV DNA (PCR)] is significantly different by gender.

The Figure 2 profile of log rel. liver HBV DNA (PCR) by treatment shows significant efficacy for the female mice at 23 mg/(kg d) DCP (*p *< .05) as compared to female controls. The figure also shows that log rel. liver HBV DNA (PCR) is significantly lower than controls for 10 mg/(kg d) adefovir dipivoxil (*p *< .05) in both sexes. Moreover, Figure 2 shows that there are significant gender differences in viral DNA levels for all DCP dosage levels tested. A similar treatment response pattern was found for the log liver HBV DNA (Southern) measure (see additional file 1: "Summary Statistics").

**Figure 3:**
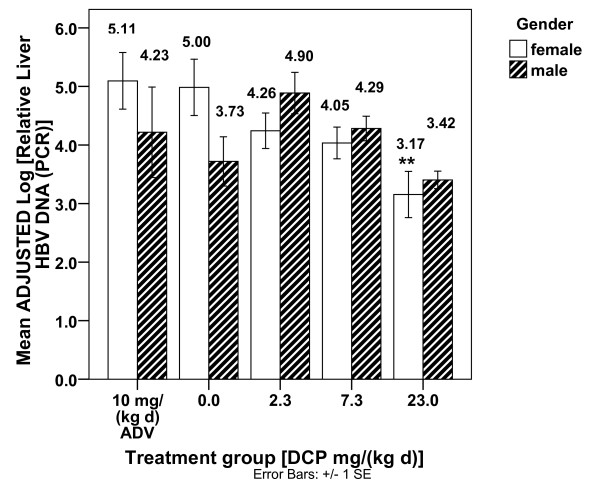
**The ADJUSTED Log [rel. Liver HBV DNA (PCR)] by treatment group and gender calculated for each HBV mouse**. In order to isolate the effects of DCP treatment away from the effects of the other DCP-EM variables, **ADJUSTED **Log [rel. Liver HBV DNA (PCR)] values were calculated for each mouse, based upon the Table 1, Table 2 regression model algebraically rearranged as follows: The **ADJUSTED **scores are graphed by treatment group and gender for the HBV mice (ADV = Adefovir dipivoxil). ** (*p *< .01) - Mean ADJUSTED Log [rel. Liver HBV DNA (PCR)] is significantly different than controls [DCP = 0.0 mg/(kg d) of the same gender] only for females at 23.0 mg/(kg d) DCP. No significant gender differences were found for any of the treatment groups (*p *≥ .095).

In order to explain the Figure 2 DCP/gender differences, an ADJUSTED log rel. liver HBV DNA (PCR) was calculated for each mouse based upon the DCP-EM regression from Table 1, Table 2, rearranged algebraically as shown in the legend of Figure 3. The resultant ADJUSTED log rel. liver HBV DNA (PCR) values demonstrated no significant gender differences among the mice for any treatment group. Therefore, the log rel. liver HBV DNA (PCR) gender differences were accounted for by adjusting for the Kyn/Trp, MCP-1, and GM-CSF serum levels for each mouse, as shown in the re-plotted ADJUSTED log rel. liver HBV DNA (PCR) scores in Figure 3.

## Discussion

The significant Table 1, Table 2 linear stepwise regression quantifies a DCP Effects Model (DCP-EM) for the HBV mice showing a significant relationship between the inhibition of hepatitis B DNA replication and DCP dosage in the context of three serum immunological changes. The three immunological changes found to be significantly associated with DCP's reduction of viral DNA levels are: 1) decreased MCP-1; 2) decreased Kyn/Trp (an estimation of IDO activity); and 3) increased GM-CSF. Among female HBV mice, 23 mg/(kg d) DCP was found to reduce control values of log rel. HBV DNA (PCR) to levels comparable to those achieved by the antiviral agent adefovir dipivoxil (Figure 2). When serum Kyn/Trp (IDO activity), MCP-1, and GM-CSF levels were taken into account and ADJUSTED log rel. liver HBV DNA (PCR) values calculated for each mouse (Figure 3), gender differences in log rel. HBV DNA levels vanished, implicating the involvement of these immunological variables in the gender differences.

Table 1, Table 2 shows that DCP decreases MCP-1 serum levels. MCP-1 (Monocyte chemotactic protein-1) is a small cytokine belonging to the CC chemokine family, also known as Chemokine (C-C motif) ligand 2 (CCL2), which recruits monocytes, memory T cells, and dendritic cells to sites of tissue injury and infection [22,23]. A number of genetic loci have been proposed to be associated with persistent hepatitis B virus (HBV) infection [24], including the MCP-1 -2518 G/G genotype and G allele, more frequent in HBV patients than controls (P < 0.01, after multiple corrections Pc < 0.05). In other studies with HBV-associated hepatoma cells, MCP-1 is found to be overexpressed [25]. Table 1, Table 2 also shows that DCP increases GM-CSF serum levels. GM-CSF induces the differentiation of granulocyte, macrophage, and eosinophil precursor cells, as well as the proliferation of monocyte-macrophages, T lymphocytes, keratinocytes, and endothelial cells. GM-CSF is produced by T lymphocytes, macrophages, and several cell types in extramedullary sites, where it may act in a paracrine manner to regulate the local response to antigenic challenge [26]. Clinical trials evaluating the efficacy and safety of GM-CSF as an adjuvant to hepatitis B vaccine in patients with end-stage renal disease showed improved seroprotection rates with HBV vaccine after GM-CSF administration [27]. Therefore, serum MCP-1 is a HBV risk factor decreased by DCP, while GM-CSF, whose serum level is increased by DCP, has been shown to be a effective HBV vaccine adjuvant.

The inhibition of the enzyme IDO by DCP is significantly correlated to the decrease of HBV DNA in the DCP Effects Model (Table 1, Table 2). Notably, the mean control value for female HBV mouse serum Kyn/Trp (22.2 ± 2.2 uM/mM) is greater than the mean control serum Kyn/Trp for male HBV mice (12.8 ± 1.1 uM/mM). Furthermore, the mean female HBV mouse Kyn/Trp value is closer in magnitude to normal human serum Kyn/Trp (26.5 - 45.0 uM/mM) [14,28,29]. These findings, taken together with the finding that the serum IDO gender difference for the HBV mice accounts, in part, for the gender differences seen in the Figure 2 DCP dose-response diagram, and strongly suggests that DCP has greater anti-HBV efficacy in female HBV mice partly because to their elevated serum IDO.

One could argue that the lower IDO activity in males would render this pathway more susceptible to the effects of DCP, and that a lower dose of this compound would be effective in targeting the more limiting IDO metabolites in male mice. However, since indoleamine 2, 3-dioxygenase (IDO) metabolizes the essential amino acid tryptophan in mammals, catalyzing the initial and rate-limiting step in the de novo biosynthesis of nicotinamide adenine dinucleotide (NAD) [30], excessively low levels of IDO, and consequently NAD, would be debilitating to the male mice. Males likely have metabolic mechanisms to prevent the IDO/NAD pathway from dropping below basal levels.

The observation that female transgenic mice have significantly higher serum IDO activity than the male mice is biologically relevant to HBV expression in these transgenic mice in the following ways. IDO immuno-inhibition has been found to take two forms, 1) as a depletor of the nutrient tryptophan, and 2) through the direct action of its enzymatic products, kynurenine, 3-hydroxykynurenine, and 3-hydroxyanthranilic acid on immune cells [10,12,14,31,32]. In previous studies, female HBV mice were identified to have slightly lower levels of liver and serum HBV DNA than male mice [33,34]. Thus, these two gender-differentiated measures, liver HBV DNA levels and IDO activity, might be connected mechanistically. For example, HBV surface antigens can be regulated in HBV transgenic mice by sex steroids and glucocorticoids [35]. Similarly, both IDO, and its related hepatic enzyme TDO (hepatic tryptophan pyrrolase; tryptophan 2,3-dioxygenase) can be upregulated by steroids like estrogen [36-38]. Therefore, there are links between the lower levels of liver HBV DNA in female mice, and hormone-regulated tryptophan metabolism. Further evidence for a gender-HBV linkage is that hepatocellular carcinoma (HCC), one of the human cancers etiologically related to HBV, affects males in a significantly higher proportion than females [39]. A similar gender effect is seen in HBV-transgenic C57BL/6 mice [40]. Also, male gender is a predictor of poor response to IFN-α treatment of chronic hepatitis B (CHB) [41].

The involvement of IDO activity in determining the levels of liver HBV DNA can explain the mechanism of the anti-HBV activity of DCP. Since DCP is known to inhibit IDO in human PBMCs [3], the higher IDO activity found in the sera of the female HBV mice allows for a proportionally greater degree of IDO inhibition by DCP, and consequently relatively greater immuno-enhancement, since IDO is a Th1-inhibitory enzyme. A higher IDO activity in the female HBV mice provides more metabolic targets for inhibition by DCP, and a greater de-repression of Th1 immune responses. In humans, altered IDO-related serum metabolite concentrations, i.e., lower tryptophan and kynurenine levels, have been found in young adult females as compared to young adult males [42].

The serum IDO differences between humans and mice might also be related to iNOS differences because 1) human macrophages are deficient in high output NO production [43,44], and 2) NO interferes with IDO expression and function [45]. Thus, the lower serum Kyn/Trp (*i.e*., lower IDO activity) in mice as compared with humans might be due to the higher NO levels in mice. Further examination of these possible factors influencing serum IDO is of interest since endogenous IDO levels are found to play a significant role in the antiviral activity of the immunomodulator DCP.

DCP inhibition of serum IDO as part of its anti-HBV effect reduces circulating kynurenine levels, and increases tryptophan availability, both of which enhance T cell-, B cell- and NK-mediated immunity [10,12,14,31,32]. Studies linking the collapse of HBV-specific CD8 T-cells, and impaired innate immunity (NK cells) in persons with chronic hepatitis B (CHB) have been reviewed [46]. HBV-specific CD8^+ ^Tcells are associated with viral control (HBV DNA load) [47,48], and reducing HBV replication to <10^7 ^copies/ml is suggested to maximize the chances of vaccines to expand a broad repertoire of HBV-specific CD8^+ ^cells [48]. Our data shows that DCP significantly inhibits IDO in the HBV transgenic mice. Moreover, a number of studies show a significant positive linkage between IDO and the PD-1/PD-L1 pathway [49-54]. PD-1 receptors are found on activated T cells, B cells, macrophages, and DCs [55], and expansion of the PD-1 pathway by T cells was associated with viral replication in a model of chronic HBV infection [56]. Therefore, a DCP-driven decrease of IDO activity should, in turn, downgrade the PD-1 pathway, expand HBV-specific T cells, and lead to a decrease in HBV DNA. Further study into these connections can more firmly establish this model.

A recent review of the woodchuck hepatitis virus model [57] discusses immunotherapeutic approaches based upon the reconstitution of Th1 immune responses. IFN-γ antiviral immune responses stimulate IDO activity in a self-limiting manner due to the T cell inhibitory effects of the IDO-produced kynurenines. By inhibiting IDO with inhibitors such as DCP, the Th1 type immune response can be enhanced while the negative effects of kynurenine are decreased. However, while IDO inhibition probably improves immune functioning, it may also serve to enhance the rate of viral reproduction due to the inhibition of tryptophan deprivation and consequent enhancement of growth conditions. On the other hand, it is also possible that some of the immunological effects of DCP are due to a general growth inhibition that diminishes virus multiplication. Also, the present study did not measure the plasma levels of HBV DNA, and the HBs and HBe antigens, which would be critical for the evaluation of the effects of DCP upon circulating HBV titers, viral assembly, and infectivity.

## Conclusions

A linear stepwise regression model, the DCP Effects Model (DCP-EM), was generated from viral and immunological measures collected from hepatitis B virus (HBV) transgenic mice treated with dipterinyl calcium pentahydrate (DCP). Three significant serum changes were found to be associated with the inhibition of HBV DNA (PCR) replication by DCP. These immunological changes were: 1) decreased MCP-1; 2) decreased Kyn/Trp (an estimation of IDO activity); and 3) increased GM-CSF. Among the female HBV mice, 23 mg/(kg d) DCP was found to reduce log HBV DNA to levels comparable to those achieved by the antiviral agent adefovir dipivoxil.

## Competing interests

PM holds stock, and PM and DF hold stock options, in SanRx Pharmaceuticals, Inc., which has been assigned patent rights to DCP.

## Authors' contributions

JM carried out the HBV transgenic mouse and virological studies, and collaborated in the drafting the manuscript. DF carried out the serum tryptophan and kynurenine measurements, and also collaborated in the drafting the manuscript. PM participated in the design of the study, carried out the statistical analyses, and was primarily responsible for drafting the final manuscript. All authors have read and approved the final manuscript.

## Supplementary Material

Additional file 1 **Summary Statistics**. The mean and standard error of each measured variable by treatment and gender. Click here for file
